# Exposure to natalizumab during pregnancy and lactation is safe – No

**DOI:** 10.1177/1352458520917934

**Published:** 2020-06-08

**Authors:** Laura Airas

**Affiliations:** Division of Clinical Neurosciences, Turku University Hospital, Turku, Finland; Department of Clinical Medicine, University of Turku, Turku, Finland/Turku PET Centre, Turku University Hospital and University of Turku, Turku, Finland

Natalizumab is a humanized monoclonal IgG4 monoclonal antibody (mAb), which binds to the α4 integrin chain of the very late antigen-4 (VLA-4) molecule. VLA-4 is expressed by T and B cells, eosinophils and monocytes. It functions as an adhesion molecule, that is, it mediates attaching of cells to other cells, or rolling of cells on other cells by binding to vascular cell adhesion molecule-1 (VCAM-1). VLA-4 is also a matrix receptor, that is, it can mediate binding of cells to fibronectin in the extracellular matrix.^1^ VLA-4 mediates adhesion of circulating leukocytes to endothelial cells and thereby their extravasation, but it also promotes adhesion of hematopoietic cells within the lymphoid tissues and controls their maturation and differentiation.

Natalizumab treatment has been available for MS patients for 14 years, during which time as many as 187,000 MS patients have been exposed to the drug. Performance of the drug in terms of keeping relapses and new inflammatory lesions at minimum is superb, but it’s cessation frequently leads to rebound activation of the previously active MS disease, which may have deleterious consequences for the patient. Interestingly, during pregnancy the rebound activation seems to be particularly frequent and severe.^2^ Moreover, natalizumab impairs the function of the immune system, and patients receiving this drug may be prone to opportunistic infections. Careful consideration regarding the use of natalizumab in the context of pregnancy and lactation is thus necessary for ensuring best possible health for both mother and the infant. The following five aspects should be considered when contemplating natalizumab, pregnancy and lactation.

## Effect of natalizumab exposure on embryonic development

In addition to their important role in the immune system, α4 integrins are also active during embryonic development. Both VLA-4 and VCAM-1 molecules are involved in placental and umbilical cord generation, and (recessive) gene disruption of the *α4* chain has been shown to have lethal consequences during embryogenesis.^1^ Immunohistochemical studies demonstrate that the α4 integrin subunit is expressed in a number of embryonic tissues including somites, neural crest, heart, smooth and skeletal muscle and other tissues, suggesting multiple roles of α4 integrins in embryogenesis.^3^ The α4-null embryos show distinct defects in two cell-cell adhesion events; allantois-chorion fusion during placental development (relevant for umbilical cord generation), and epicardium-myocardium attachment during cardiac development.^1^ Exposure of rat embryos to anti-VLA-4 antibodies or VLA-4 antagonists similarly led to defects in allantois-chorion fusion.^4^ Hence, disturbing normal function of VLA-4 during early human development may have deleterious and widely varied effects for the embryo. This should be taken into account when considering natalizumab use during pregnancy, although it is presently still quite uncertain at what level the circulating natalizumab mAb has access to the early developing human embryo.

The available human reports and studies regarding natalizumab and reproductive safety are underpowered to draw firm conclusions for rare events. The largest published study was performed by Biogen (the manufacturer of natalizumab). It used a pregnancy registry with information from patients exposed to the drug at any time within 3 months prior to conception or during pregnancy. The results from the register were not entirely straightforward.^5^ Foetal outcome information was available from 317 pregnancies, and 9.2% of the newborns had a congenital anomaly. In 5.7%, the congenital defect was considered to be major, that is, there was an abnormality of structure, function or metabolism that was either fatal or resulted in physical or mental disability. The overall rate of major birth defects in the registry was higher than the rate of 2.7% of major malformations reported by the Metropolitan Atlanta Congenital Defects Program (MACDP), or the European Concerted Action on Congenital Anomalies and Twins (EUROCAT) register (2.3%). No specific pattern of birth defects was observed. When comparing the major malformation frequency of the natalizumab pregnancy register to the MACDP numbers, the number needed to harm (NNH) for natalizumab exposure is 41. Compared to the EUROCAT register the NNH is 36. Regrettably, this register has been terminated and is not collecting more cases.

## Effect of natalizumab cessation on MS disease activity

Many of natalizumab-treated MS patients are young fertile women with aggressive disease. Among such patients, withdrawal of the drug may result in severe disease reactivation, and hence cessation of natalizumab should be planned well ahead of a potential pregnancy to avoid a severe rebound disease during pregnancy.^2^ During early pregnancy, mother’s immune cells acquire a pro-inflammatory phenotype, which is considered necessary for normal physiological development of the placenta.^6^ This physiological phenomenon may well predispose the mother to a more severe rebound activation of the disease in early pregnancy upon cessation of natalizumab. Regarding the above-discussed role of α4 in embryogenesis, continuing natalizumab into early pregnancy causes an unnecessary risk for foetal development. Stopping the drug shortly before pregnancy, on the other hand, predisposes the mother to a severe rebound disease activation during pregnancy. This implies that pregnancy-planning should take place early, and potentially a bridging therapy with another drug with more favourable features regarding pregnancy should be considered.

## Effect of natalizumab on infection risk during pregnancy

The function of mother’s immune system is modulated during pregnancy to ensure normal development of the foetus, and in mid-pregnancy the T-cell-mediated adaptive immune responses are suppressed, and this usually results with amelioration of Th1-type autoimmune diseases.^6^ The altered T cell function might render the mother more susceptible to certain infections, such as herpes virus infections or progressive multifocal leukoencephalopathy.^7^ If natalizumab is used during pregnancy, this might further impair mother’s immune protection towards these pathogens.^8^

## Effect of natalizumab on the development of the foetal hematopoietic system

There are case series reporting accidental natalizumab use throughout pregnancy.^9^ Natalizumab, similarly to other IgG immunoglobulins, passes readily through the placenta into the foetus during the third trimester of pregnancy. In cases exposed to natalizumab in late pregnancy, haematological abnormalities including thrombocytopenia and anaemia was observed in the majority of the infants, some with related adverse effects such as intracerebral haemorrhage or hypoxia.^9^ Finally, newborn’s immune system is still under-developed at time of birth, and it is conceivable that exposure to natalizumab may further impair it’s function and render the baby even more susceptible to infections. Long-term follow-up of these babies are yet to be reported.

## Effect of natalizumab-containing *breastmilk* on the newborn

Breastfeeding is recommended to boost infant’s immune function, and to provide optimal nutrition and bonding between mother and newborn. Occasionally, MS disease rebound is observed after the delivery, and decisions need to be taken regarding treatment and breastfeeding. Majority of the immunoglobulins in human breastmilk are of IgA class, but it is now known that also IgG class immunoglobulins are present, and possibly absorbed through infant’s gut into the circulation via the neonatal Fc receptor.^10^ Interestingly, accumulation of natalizumab was shown in human breastmilk after repeated natalizumab infusions, and thus safety of natalizumab treatment during breastfeeding could not be established.^10^ Due to the beneficial effects of breastfeeding to the infant, decisions about cessation of breastfeeding should not be taken lightly, and drugs with better established safety during breastfeeding should be considered to keep mothers MS disease under control while breastfeeding.

In conclusion, information on natalizumab-therapy in association with pregnancy is still too scarce to recommend treatment continuation during pregnancy. Following the last infusion, a 2- to 3-month wash-out period before omitting contraception should ensure reproductive safety. This clearly causes problems related to potential rebound disease activity, and a bridging therapy to keep MS disease under control before and during pregnancy may be warranted. Pregnancy-planning may be challenging at times, and accidental pregnancies during natalizumab treatment are unavoidable. In these instances, enhanced antenatal and post-natal foetal monitoring should take place.
